# Factors contributing to medicines wastage in public health facilities of South West Shoa Zone, Oromia Regional State, Ethiopia: a qualitative study

**DOI:** 10.1186/s40545-019-0192-z

**Published:** 2019-11-11

**Authors:** Esayas Tadesse Gebremariam, Dawit Teshome Gebregeorgise, Teferi Gedif Fenta

**Affiliations:** 1 Department of Pharmacy, Unit of Pharmaceutics and Social Pharmacy, College of Medicine and Health Sciences, Ambo University, P.O. Box: 19, Ambo, Ethiopia; 2 Department of Pharmaceutics and Social Pharmacy, School of Pharmacy, College of Health Sciences, Addis Ababa University, P.O. Box: 1176, Addis Ababa, Ethiopia

**Keywords:** Wastage, Contributing factors, Medicines wastage, Public health facility, Ethiopia

## Abstract

**Background:**

Medicines wastage is one of the challenges of health supply chain management in developing countries including Ethiopia. However, there is lack of objective evidence on the detailed underlying causes of medicines wastage. Therefore, the aim of this study is to explore factors contributing for medicines wastage in selected public health facilities of South West Shoa Zone, Oromia Regional State, Ethiopia.

**Methods:**

A qualitative study was conducted in 10 public health facilities (1 General hospital and 9 health centers). An in-depth interview with flexible probing techniques was employed to collect the data from 20 key informants from May 2, 2016 to May 27, 2016. A semi structured interview guide was prepared to explore key informants’ idea about current situation of contributing factors and efforts made to reduce medicines wastage in public health facilities. A thematic analysis was then used to analyze the data.

**Results:**

Almost all key informants felt that medicines wastage is increasing from time to time in their health facility due to supplier’s issuing of medicines without health facilities’ needs and request, failure to follow first expired first out principle in issuing medicines from stores to different dispensing units, lack of communication between supplier and health facilities, inadequate number of pharmacy personnel and weak monitoring system of supply chain in the health facilities. They also revealed budget constraint, stock out of medicines and congested store are of the major challenge that are compromising their service provision.

**Conclusion:**

Medicine wastage is an ever-increasing problem in public health facilities of South West Shoa Zone. The problem is exacerbated due to absence of mechanisms to minimize the wastage. Health facilities, therefore, should place a mechanism to exchange medicines from overstocked facilities to under stocked ones; has to improve store management capacity by employing competent professionals, equipping the store with necessary technology and introducing a monitoring and evaluating health supply chain performance system in the health facilities.

## Introduction

Medicines are key elements of a well-functioning healthcare system. However, one third of the global populations do not have regular access to medicines. The extent of the problem is even worse in some of the lowest-income countries in Africa and Asia where more than half of the populations have no regular access to essential medicines [1]. One of the reasons for such high inaccessibility of medicines is wastage [2].

According to World Health Organization (WHO), medicines wastage is defined as unwanted medications which include expired, unused, spilt and contaminated pharmaceutical products, drugs, vaccines and sera that are no longer required and need to be disposed of appropriately [3]. In this study, medicine wastage indicates medicines expired, damaged, lost, obsoleted and unsafe for use.

Medicines wastage not only hampers therapeutic benefit but it also affects financial capability. On average, countries spend about 25% of their total health expenditure on medicines. Of these, according to Management Science for Health estimation, 70% of the total funds invested on essential medicines are wasted in normal supply system [4].

Factors contributing towards medicine wastage are diverse [5]. In developed countries, death of a patient, switching one medicine to other, discontinuation of treatment, side effects and poor patient compliance were reported as contributing factors of medicines wastage [6].

In low income countries, although factors associated with medicine wastage in health care settings are not well documented [5], a report shows that weak supply chain management system takes the lion share [7]. This includes selection and quantification of medicines without proven data/evidence and techniques which could lead to overstocking/under stocking of the medicines. In addition, poor storage conditions such as direct storage of medicines on the floor; lack of systematic arrangement of stock; presence of dust and pests; inadequate protection from direct sunlight; and lack of provision of temperature monitoring charts and facilities to monitor room temperature could lead to degradation of medicines. Moreover, poor inventory management, which is estimated to cause for the wastage 4 to 9% of medicines in overall supply systems, is identified as contributing factors [4, 8].

Studies conducted in Uganda and Tanzania also revealed supply chain related factors are main contributing factors for medicines wastage. Some of these are irrational procurement practice, supplying medicine without the need and request of a client, neglecting stock monitoring, lack of knowledge on expiry prevention tools, non-participation of clinicians in medicine selection and quantification in hospitals, profit- and incentive-based quantification, third party procurement by vertical programmes and overstocking [9, 10]. In Tanzania, over stocking and pilferage were identified as the two top factors contributing to medicines wastage [5].

In Ethiopia, all public health facilities are required to buy medicines from single public supplier. Public health facilities can only buy medicines from private sector when a public supplier declares stock out or the medicine is not imported by the agency. Each year, while hospitals enter contract with a supplier by themselves, health centers enter agreement with a supplier through the health bureaus. In the contract, both parties agree on the type and amount (by value) of medicine they would buy and supply. Once a transaction is processed, the finance department of the respective hospitals/health centers deposits the money to supplier account. There are some which deposit the money to supplier account in advance [11].

Like many other African countries, medicines wastage is one of the challenges of the health supply chain management in Ethiopia [12]. For instance, in 2003, 8% of the total medicines were expired [13]. Poor documentation, absence of accountability, lack of software/tools that automatically capture data and absence of a system that oblige health facilities to document and report wastage to the immediate concerned body in the supply chain are some of the factors contributing to medicines wastage at the different levels of the health supply chain [2].

In South West Shoa Zone, we have seen quite a large proportion of space at the medicines store is occupied by expired and unfit for use medicines. However, the underlying causes for this were not explored. Although there are reports that reveal the presence of wasted medicines in Ethiopia, they lack objective evidence on the detailed underlying causes of medicines wastage. Therefore, the aim of this study was to explore factors contributing to medicines wastage in selected public health facilities of South West Shoa Zone, Oromia Regional State, Ethiopia.

## Methods

A qualitative study employing an in-depth interview with flexible probing techniques was employed to collect data from the key informants in the selected public health facilities. All Chief Executive Officers (CEO) and Heads of Pharmacy case teams in the selected health facilities of South West Shoa Zone were interviewed.

The numbers of health facilities to be included in the study were calculated by using the Logistics Indicators Assessment Tool (LIAT). This document suggests that at least 15% of the target health facilities should be selected as a sample for conducting such study [14]. At the time of survey, there were 1 public hospital and 55 health centers (10 Type A and 45 Type B) which were providing service in the zone. Of these, the hospital was selected purposively; and 9 health centers (two type A and seven type B health centers which vary based on their patient load and closeness to urban areas) chosen by using simple random sampling techniques to get maximum variation. Accordingly, 10 CEOs and 10 heads of pharmacy case teams (a total of 20) were purposively identified as key informants for the study because they are supposed to have rich information other than health professionals.

A semi structured interview guide was prepared to explore key informants’ idea about current situation of contributing factors and consequences of medicines wastage in the selected health facilities. The interview guide was developed based on previous literatures [2, 5, 9, 10] and this was tested for its face and content validity by two experts from Social and Administrative Pharmacy Research group. The interview guide was prepared in English, translated into Amharic and back translated to English by two authors (ETG and DTG) to check message consistency. All the interviews were conducted by the principal investigator in Amharic to facilitate conversation and not to be limited by language barrier. On average, the interview took 40 min and was held in a private setting. All the interviews were tape recorded and transcribed verbatim.

The data collection was carried out from May 2 to May 27, 2016. The data analysis involved an intensive reading by two authors (ETG and DTG) in order to identify key themes. Audio-recorded interviews were transcribed verbatim and the raw data was categorized under pre-developed coded themes and sub themes. A thematic analysis was then used to analyze the data. Initial categories for analyzing data were drawn from the interview guide and themes and patterns emerged after reviewing the data. Key themes to emerge were: situation of medicines wastage; contributing factors and consequences medicines wastage in the health facilities. Then data was handled manually.

Ethical approval was obtained from the Ethics Review Board of the School of Pharmacy, Addis Ababa University; South West Shoa Zone Health Department and from the respective health facilities. Besides, a verbal consent was obtained from all participants before starting the actual data collection. Confidentiality and anonymity of the information was maintained by avoiding any personal identifiers in the data presentations.

## Results

In-depth interviews were held with CEOs and heads of pharmacy case teams. Except one, all the key informants were males who were in the age group of 30 to 35 years. Their work experiences ranged from 1 to 7 years (Table 1).

**Table 1:** Socio demographic characteristics of key informants working in the selected public health facilities in South West Shoa Zone, Ethiopia, May 2016 (*n* = 20)

Socio-demographic Profile	Number
Gender	
Male	19
Female	1
Age	
25–29	8
30–35	11
≥ 35	1
Profession	
General practitioner	1
Pharmacist	7
Druggist	3
Health officer	4
Nurse	5
Level of education	
Diploma	6
Degree	14
Work experience	
< 5 years	13
5–10 years	7

A pattern of responses emerged from the interviews in three themes. These are situation of medicines wastage, factors that contribute for medicines wastage and the consequence of medicines wastage on service provision.

### The situation of medicines wastage in public health facilities

Almost all key informants said medicines wastage is a problem in their facility but they differed in the trend. While some said it is increasing over time, others said the opposite.

One respondent said:

*“Medicines wastage is decreasing overtime due to the advent of Integrated Pharmaceutical Logistic System/IPLS in recent years.” (Pharm.02)*

In contrast, another respondent said,

*“ … . Medicine wastage is increasing overtime since we are receiving medicines from central/regional medical store based on ‘Push’ system.” (Pharm.03)*

### Factors contributing for medicines wastage

Key informants cited supplier and public health facilities related factors as reasons for medicines wastage (Table 2). A statement made by one key informant demonstrated this fact:

*“Medicines wastage in our facility is a result of internal (e.g. shortage of staffs and lack of administrative support) and external factors (e.g. Supplier challenges)” (Pharm.04)*

Supplier related factors includes, among others, issuing of medicines without health facilities’ needs and requests, delivery of near expiry medicines, lack of communication between supplier and health facilities; and having weak supply chain monitoring system.

Provision of medicines not based on needs and request and delivery of near expired medicines by supplier are major causes of this problem. One respondent illustrated the situation as follows:

*“If I refuse to receive a near expire medicines, I will not able to get other required medicines listed in the same voucher … [So,] I will be forced to receive the medicines, knowing that it expires before it is consumed.” (Pharm.03)*

Another one adds on this:

*“Our supplier does not usually provide us the medicines as per the requested type and quantity. For example, we recently received several tins of phenobarbital that we had not ordered. Most of the time they do this to balance/match the budget we have with the value (financial) of products they issue. They themselves adjust the quantity and provide us overstock medicines.... such practice led to medicines wastage.” (Pharm.07)*

There are also cases that supplier issue near expiry medicines to the health facilities so as to transfer the blame for medicine wastage from supplier to health facilities and in order not to be rated low in their performance.

In the health facilities, not using FEFO for issuing medicines from stores to different departments, and lack of communication between different dispensing units were identified as contributing factors for medicines wastage.

*“I think the reasons for expiry in our health center are lack of communication within different dispensing units in the health center and not prioritizing based on expiry date status upon issuing items from store to different units.” (Pharm.04)*

Shortage of pharmacy professionals was also mentioned as a reason. In some facilities, due to high pharmacy professionals’ work load, clinical nurses are managing the supply for which they are not trained. This contributes a lot for wastage of medicines. One key informant said that

*“Because of shortage of pharmacy professionals in the facility, we (pharmacy professionals) are not focused on medicines management. As a result, clinical nurses are taking care of supply chain activities such as managing store in addition to their routine nursing functions. Clinical nurses lack training in medicines logistics management.” (Manager.01)*

Some key informants mentioned health facilities’ management negative attitude and weak support to the pharmacy service as contributing factors for medicines wastage. They also added lack of reporting and auditing, absence of accountability for wasted medicines and lack of regular discussion with key stakeholders on wastage and related issues. One key informant said that

*“For me, administrators of the health facility have no good attitude to the pharmacy service. When we (pharmacy professionals) propose something to improve the medicine supply, we will not get support from the management. I think this has its own contribution on medicines wastage.* ” *(Pharm.05)*

**Table 2:** Key drivers of medicines wastage in selected public health facilities of South West Shoa Zone, Ethiopia, May 2016

S.no	Contributing Factors
1	Near expiry medicines (< 6 months) are being delivered to the health facilities by the supplier
2	Poor stock management, e.g. not applying FEFO principle in stock management
3	Lack of electronic stock management tools that are used to trace expiry date in the health facilities
4	Shortage of pharmacy professionals in the facilities
5	Health facility administrators’ negative to the pharmacy service
6	Weak or no mechanisms for medicine wastage monitoring and evaluation in the health facilities
7	Poor communication and coordination between the health facilities and key stakeholders (health bureau/office, suppliers and donors)
8	Poor storage conditions of medicines in the health facilities

### The consequence of medicines wastage on service delivery

When asked about the consequences of medicines wastage on service provision, key informants said it caused congested store, financial burden (budget constraint) and stock out of medicines.

One respondent said:

*“Expired and damaged pharmaceuticals congested our storage and occupied our limited space that could be used for other medicines. So, patients cannot be assured that they are receiving a high quality medicine because these items are a result of poor storage conditions.” (Pharm.01)*

## Discussion

Majority of key informants felt that medicines wastage is increasing from time to time in their facilities. Some also said it is decreasing compared to the past due to strong implementation of IPLS in recent years. Impact evaluation report showed that IPLS is reducing wastage of medicines [15].

The current study found that provision of medicines without needs and requisition, poor stock rotation/ not using FEFO for issuing medicines from stores to different dispensing units, lack of communication between supplier and health facilities, between dispensing units within a health facilities, inadequate pharmacy professionals and weak medicines wastage monitoring system as major contributing factors for wastage. Previous studies conducted in Uganda and Ethiopia also reported the same where short shelf life, poor forecasting of need, poor storage practices and poor inventory control were identified as major factors for high medicines wastage [9, 15]. In contrast to the above findings, studies conducted in developed countries showed that poor medicine compliance, discontinuing or switching of medicines, side effect and patient’s death are contributing factors for medicines wastage [16, 17]. This could be due to differences in the settings and medicines investigated, where the former studies in developed countries assessed medicines wastage in community and medicines returned to pharmacy by patients.

In Ethiopia, the law restricts public health facilities primarily to buy from public supplier based on the agreement entered between two parties every year. Accordingly, the supplier will commit to make products available as per the contract. Unless the supplier declared stock out of medicines, public health facilities are not allowed to buy from private or other suppliers. That is why they issue near expiry medicines (< 6 months) to the health facilities. Similarly, different studies conducted in Africa indicated provision of medicines that were about to expire as a factor for its expiry [10, 18, 19]. In order supply medicines with long expiry date based on the health facilities need, ensuring data visibility at all echelon in the supply chain is crucial to make the right decision.

Lack of communication between different units in a health facility, between public supplier and health facilities; and inter-health facilities regarding consumption of medicines was mentioned as reasons for wastage in the current study. Poor coordination between key stakeholders appears to be responsible for some expiry incidents in health facilities in Uganda as well [9]. Arranging a platform that concerned stakeholders will participate to make a regular review of performance and make high level decisions should be in place. For routine activities, technologies such as group SMS texts and some apps e.g. Whatsapp, telegram could be used to avoid communication barrier, one to learn from the other and make an informed decision.

Poor stock rotation also leads to expiry of medicines. It is estimated to contribute between 4 to 9% of the overall waste in supply systems [4, 8]. Medicines with high wastage rates attributable to expiration should be checked to see if shelf life is abnormally low. If so, it should be monitored very closely and probably moved to the front of the shelf or reallocated to facilities with higher consumption rates for those products.

Shortage of pharmacy staffs was mentioned as one contributing factors for medicines wastage. This finding is consistent with reports conducted in the country where critical shortage of trained pharmacy professionals at regional and zonal level coupled with very high attrition towards the private sector was identified as an important factor that drags back the drug and supplies management component of the health sector development plan [20, 21]. Also, the result is consistent with most developing countries specially the Sub-Saharan which are suffered from pharmacist workforce disparity according to the International Pharmaceutical Federation reports [22–24].

Financial burden (budget constraint) and stock out of medicines were among the consequences mentioned by key informants. In many developing countries, budgets for medicines are often tight, and many people are unable to access essential medicines, medicine wastage reduces the quantity of medicines available to patients and therefore the quality of health care they receive [9, 25]. For instance, a report by Daily Mail Online indicated that Ethiopia to throw away 69 million ‘poor quality’ condoms that bought in $2million (£1.38million) of international aid money wasted after they fail ‘hole test’ [26]. Therefore, it is important to decrease medicines wastage to optimize over all financial loss incurred and to compromise frequent stock-out of medicines in the health facilities which in turn would have a positive implication for the achievement of health sector transformation plan target of below 2% average rate of medicine wastage in Ethiopia [12].

Furthermore, the study identified congested store due to accumulated medicine wastes that are not disposed timely as a reason. This may lead to inefficient use of storage space in health facilities, limiting available space for inventory of usable medicine supplies [9, 27]. Therefore, the facilities should find a mechanism to regularly dispose wasted medicines since it occupy limited storage areas and potentially create mix-ups.

Certain amount of medicines wastage is inevitable, but about 50% of them is likely to be preventable [16]. And all key informants suggested that having adequate number of pharmacy professionals, conducting regular supervision, having regular discussion on medicines supply management activities with key stakeholders and improving communication of the health facility with other health facilities and supplier were among the suggestions made to improve medicines wastage.

### Limitation of the study

This study did not include unused medicines in wards and at each household level. The findings of this study were based on health facilities perspective only. It did not include other stakeholders’ perspectives such as; PFSA, Oromia regional health bureau, zonal and woreda health offices and partners.

## Conclusion

Medicine wastage is a persistent problem of public health facilities in South West Shoa Zone and several reasons were pointed out as contributing factors for this problem. Deploying more pharmacy workforce, exercising proper stock management supported by technology and creating a mechanism of exchanging medicines from overstock to under stock areas should be done to minimize wastage.

## Data Availability

The data sets generated in this article are available on AAU institutional repository of Addis Ababa University in its PDF format as *Assessment of medicines wastage and its contributing factors in selected public health facilities in South West Shoa Zone, Oromia Regional State, Ethiopia* **URl:** http://etd.aau.edu.et/handle/123456789/1467, http://etd.aau.edu.et/bitstream/handle/123456789/1467/Esayas%20Tadesse.pdf?sequence=1&isAllowed=y

## Abbreviations

CEO: Chief Executive Officer
FEFO: First-Expiry-First Out
IPLS: Integrated Pharmaceutical Logistics System
LIAT: Logistics Indicators Assessment Tool
PFSA: Pharmaceutical Fund and Supply Agency
WHO: World Health Organization
